# Comparison of some secondary metabolite content in the seventeen species of the Boraginaceae family

**DOI:** 10.1080/13880209.2016.1265986

**Published:** 2017-01-31

**Authors:** Sławomir Dresler, Grażyna Szymczak, Małgorzata Wójcik

**Affiliations:** a Department of Plant Physiology, Maria Curie-Skłodowska University, Lublin, Poland;; b Botanical Garden of Maria Curie-Skłodowska University, Lublin, Poland

**Keywords:** Chemometrics, capillary electrophoresis, allantoin, rosmarinic acid, shikonin, rutin

## Abstract

**Context:** The Boraginaceae family comprises plants that have important therapeutic and cosmetic applications. Their pharmacological effect is related to the presence of naphthaquinones, flavonoids, terpenoids, phenols, or purine derivative – allantoin.

**Objective:** In the present study, comparison of some secondary metabolite content and phytochemical relationship between 17 species of the Boraginaceae family were analyzed.

**Materials and methods:** High performance capillary electrophoresis (HPCE) was used to perform a chemometric analysis in the following Boraginaceae species: *Anchusa azurea* Mill., *Anchusa undulata* L., *Borago officinalis* L., *Buglossoides purpurocaerulea* (L.) I.M. Johnst., *Cerinthe minor* L., *Cynoglossum creticum* Mill, *Echium italicum* L., *Echium russicum* J.F. Gmel., *Echium vulgare* L., *Lindelofia macrostyla* (Bunge) Popov (syn. *Lindelofia anchusoides* (Lindl.) Lehm.), *Lithospermum officinale* L., *Nonea lutea* (Desr.) DC., *Omphalodes verna* Moench (syn. *Cynoglossum omphaloides* L.), *Pulmonaria mollis* Wulfen ex Hornem., *Pulmonaria obscura* Dumort., *Symphytum cordatum* Waldst. & Kit ex Willd., and *Symphytum officinale* L.

**Results:** Six active compounds in shoot extracts (allantoin, *p*-hydroxybenzoic acid, rutin, hydrocaffeic acid, rosmarinic acid, and chlorogenic acid) and four compounds in root extracts (allantoin, hydrocaffeic acid, rosmarinic acid, and shikonin) were identified. The presence and abundance of these compounds were used for the characterization of the species and for revealing their phytochemical similarity and differentiation.

**Discussion and conclusion:** The present study provides the first comprehensive report of the extraction and quantification of several compounds in Boraginaceae species (some of them for the first time). Among the 17 species studied, species with potentially high pharmacological activity were recognized.

## Introduction

The Boraginaceae family includes approximately 2000 species worldwide, mainly in Europe and Asia. The family comprises a group of plants that are important for pharmacology and cosmetology. The therapeutic effect of these plants is related to the content of many biologically active compounds, including naphthaquinones, flavonoids, terpenoids and phenols. The constituents isolated from these plants exhibit antimicrobial, antitumor, antiviral, anti-inflammatory, cardiotonic, contraceptive and antiplatelet activity (Sharma et al. 2009; Papp et al. 2011; Taravati et al. 2014). However, these plants are also rich in hepatotoxic pyrrolizidine alkaloids and thus consumption thereof is questionable; nevertheless, the use of Boraginaceae plants as a poultice for wounds does not rise any objection. Moreover, some plants, for example *Borago officinalis* L. or *Echium vulgare* L., have been recognized as a good source of unique fatty acids γ-linolenic acid (GLA, 18:3*n*-6) and stearidonic acid (SA, 18:4*n*-3) (Velasco & Goffman 1999; Król & Kowalski 2004; Mhamdi et al. 2009). Although Boraginaceae plants exhibit beneficial features, the medical use of many of them has still been undiagnosed. In this context, the phytochemical recognition of these species appears desirable. One of the approaches of phytochemical data processing involves using multivariate chemometric methods in chemotaxonomic studies (Dresler et al. 2016). Such a strategy provides a useful tool for comparison of species showing well-known medical properties with species that have been unrecognized until now to reveal their beneficial properties. On the other hand, phytochemical studies of unrecognized species may provide knowledge about new sources of natural bioactive compounds.

In the present study, some secondary metabolites content and phytochemical relationships between 17 Boraginaceae species (*Anchusa azurea* Mill., *Anchusa undulata* L., *Borago officinalis* L., *Buglossoides purpurocaerulea* (L.) I.M. Johnst., *Cerinthe minor* L., *Cynoglossum creticum* Mill, *Echium italicum* L., *Echium russicum* J.F. Gmel., *Echium vulgare* L., *Lindelofia macrostyla* (Bunge) Popov (syn. *Lindelofia anchusoides* (Lindl.) Lehm.), *Lithospermum officinale* L., *Nonea lutea* (Desr.) DC., *Omphalodes verna* Moench (syn. *Cynoglossum omphaloides* L.), *Pulmonaria mollis* Wulfen ex Hornem., *Pulmonaria obscura* Dumort., *Symphytum cordatum* Waldst. & Kit ex Willd., and *Symphytum officinale* L.) were analyzed. The phytochemical analysis of these species was conducted in two steps. First, a traditional strategy based on qualitative and quantitative analysis of several compounds in the plant extracts by high-performance capillary electrophoresis (HPCE) was employed. In the second strategy, multivariate chemometric techniques were applied to reveal similarities and differences between the Boraginaceae species studied.

## Materials and methods

### Plant material

Fifty-one individuals of 17 species of the Boraginaceae family (Table 1) (three individuals of each species) were collected in June 2015 in the Botanical Garden of Maria Curie-Skłodowska University, Lublin, Poland (51°16′ N, 22°30′ E). The shoots and roots were carefully washed in tap water followed by distilled water and dried at room temperature for 14 days. Afterwards, the air-dry material was milled into coarse powder using a basic microfine grinder drive IKE A11 (IKE-Werke, Staufen, Germany). The powder (1.0 g) was extracted three times with a fresh portion of decreasing concentrations of methanol (100, 80, 60%) in an ultrasonic bath for 30 min at a temperature below 40 °C. The methanolic extracts were then centrifuged at 10,000 *g* for 5 min and the mixture of the three supernatants of each sample was filtered through a 0.22 μm membrane filter. Since shikonins occur only in the underground plant parts, the sample of powdered roots was divided into two parts. The first part was extracted as described above, while the other part (1.0 g) was extracted with 4 mL hexane in an ultrasonic bath for 30 min (extraction for shikonin derivatives). Afterwards, the extract was centrifuged at 10,000 *g* for 5 min and the supernatant was evaporated; next, the residue was dissolved in 2 mL of methanol and filtered through a 0.22 μm membrane filter.

**Table 1.** List of species used in the study^a^.

Genus	Species	Voucher specimen number
*Anchusa*	*azurea* Mill.	3122-E
	*undulata* L.	3662-E
*Borago*	*officinalis* L.	100/2014-S
*Buglossoides*	*purpurocaerulea* (L.) I.M. Johnst.	784-E
*Cerinthe*	*minor* L.	3559-E
*Cynoglossum*	*creticum* Mill.	3692-E
*Echium*	*italicum* L.	3699-E
	*russicum* J.F. Gmel.	2697-P
	*vulgare* L.	4366-A
*Lindelophia*	*macrostyla* (Bunge) Popov	2893-A
*Lithosperum*	*officinale* L.	267-P
*Nonea*	*lutea* (Desr.) DC.	1466-E
*Pulmonaria*	*mollis* Wulfen ex Hornem.	3629-E
	*obscura* Dumort.	1041-E
*Omphalodes*	*verna* Moench	864-E
*Symphytum*	*cordatum* Waldst. & Kit ex Willd.	896-A
	*officinale* L.	2583-P

a All plant species were authenticated by botanist Grażyna Szymczak PhD (Director of the Botanical Garden of Maria Curie-Skłodowska University, Lublin).

### HPCE analysis

The analysis of the methanol and hexane extracts was conducted using an Agilent 7100 capillary electrophoresis system equipped with a diode-array detector (UV-VIS, DAD) (190–600 nm) (Agilent Technologies, Santa Clara, CA). The separation was performed according to the modified method described previously (Dresler et al. 2015). Briefly, fused silica 50 μm i.d. and 64.5 cm total length capillaries (Agilent Technologies, Santa Clara, CA) were used. The new capillary was flushed with 1 M NaOH (at the pressure of approx. 1 bar) for 15 min, followed by water (15 min) and a background electrolyte (BGE). The BGE consisted of a 50 mM borate solution with pH adjusted to 9.2 with 1 M NaOH. The separation conditions were set at 30 kV, temperature of 27 °C, and the run time of 12 min. Before each run, the capillary was flushed with BGE for 5 min (the total analysis time was 17 min). The samples were injected under 50 mbar pressure for 4 s. The separated components were identified by comparison of their retention times and spectra in the range of 190–600 nm with standards (Sigma-Aldrich, St. Louis, MO). The detection wavelengths depended on the compound analyzed: 194 nm for allantoin and 4-hydroxybenzoic acid; 202 nm for rutin, hydrocaffeic acid, and rosmarinic acid; 218 nm for derivatives of shikonin and chlorogenic acid. The electropherograms were analyzed using Agilent 3D-CE ChemStation software (Agilent Technologies, Santa Clara, CA).

### HPCE data analysis

Based on the HPCE data, the content of the analyzed compounds was calculated. Additionally, the HPCE data of each electropherogram were exported into a numeric format for further multivariate non-target polar compounds analysis. First, the data matrix obtained was subjected to the correction process according to the modified equation (Dresler et al. 2016):

$$\mathrm{Corrected}\;\mathrm{absorbance}=\frac{\mathrm{Absorbance}}{60\times \mathrm{migration}\;\mathrm{time}}$$

Afterwards, the alignment procedure was carried out with the Recursive Alignment by Fast Fourier Transform (RAFFT) method with the max shift parameter set at 10 (Jiang et al. 2013) using SpecAlign software (SpecAlign ver. 2.4.1, University of Oxford). Before the chemometric analysis, the average electropherograms for each species were constructed based on the analysis of three individuals per species. The phytochemical relationship between the species was visualized as a dendrogram constructed using hierarchical clustering methods with a Pearson correlation distance matrix. The calculations were performed using Statistica ver. 6.1 software (StatSoft, Inc. 2004 Statistica, Tulsa, OK). 

## Results and discussion

The phytochemical profile of each Boraginaceae species was analyzed in two ways. The first traditional approach was based on integration and identification of selected peaks on the HPCE electropherograms; in the other one, the chemometric methods were used to construct a profile of each species and compare it with the other species. Such an approach, on the one hand, provided quantitative information about the compounds; on the other hand, the chemometric methods applied revealed hidden trends and pattern recognition in the multivariate data obtained (Gad et al. 2013).

Based on the migration time and UV-VIS absorption spectrum similarity (above 99.99%, 190–600 nm) to chemical reference standards, we identified and quantified several biologically active compounds: six compounds in the shoots (rosmarinic acid, chlorogenic acid, *p*-hydroxybenzoic acid, rutin, allantoin, hydrocaffeic acid) and four compounds in the roots (rosmarinic acid, allantoin, hydrocaffeic acid, shikonin). Rosmarinic acid and allantoin were present in all the species studied (allantoin was not detected in the shoots of *A. azurea* and in the roots of *B. officinalis*), whereas the other compounds were present in a detectable quantity only in some species (Table 2). In spite of the ubiquity of allantoin and rosmarinic acid in the plants studied, the concentrations of these compounds differed significantly depending on the plant species and organ. They were in the range of 0.6–11.8 and 1.2–36.6 mg g^−1^ air-dry matter in the shoots and 0.1–34.9 and 1.3–17.0 mg g^−1^ air-dry matter in the roots for allantoin and rosmarinic acid, respectively. The content of the other identified compounds was in the range of 0.17–0.20, 0.27–0.75, 0.14–1.58 and 0.36–1.03 mg g^−1^ air-dry matter for p-hydroxybenzoic acid, rutin, hydrocaffeic acid and chlorogenic acid, respectively, in the shoots, and 0.09–0.21, and 0.07–0.28 mg g^−1^ air-dry matter for hydrocaffeic acid and the sum of shikonin derivatives, respectively, in the roots.

**Table 2.** Average concentration (±SD) of biologically active compounds in selected species of the Boraginaceae family (*n* = 3).

	Shoots (mg·g^−1^ air-dry matter)						Roots (mg·g^−1^ air-dry matter)			
	Allantoin	PHBA	Rutin	Hydrocaffeic acid	Rosmarinic acid	Chlorogenic acid	Allantoin	Hydrocaffeic acid	Rosmarinic acid	Shikonin
*Anchusa azurea*	nd	nd	nd	nd	24.0 ± 9.3	nd	0.21 ± 0.11	nd	3.5 ± 0.62	nd
*Anchusa undulata*	2.44 ± 0.41	0.183 ± 0.034	0.457 ± 0.055	nd	25.9 ± 1.4	nd	0.08 ± 0.13	nd	27.0 ± 5.65	nd
*Borago officinalis*	0.83 ± 0.27	nd	nd	nd	3.7 ± 0.2	nd	nd	nd	9.2 ± 1.16	nd
*Buglossoides purpureocaerulea*	2.93 ± 0.81	nd	0.273 ± 0.001	nd	33.1 ± 2.8	0.368 ± 0.03	1.86 ± 1.31	nd	2.0 ± 0.43	nd
*Cerinthe minor*	2.83 ± 0.51	nd	nd	1.578 ± 0.332	15.9 ± 0.7	nd	0.21 ± 0.12	nd	4.9 ± 1.33	nd
*Cynoglossum creticum*	0.67 ± 0.21	nd	0.575 ± 0.100	0.902 ± 0.029	9.6 ± 2.2	nd	0.66 ± 0.64	0.126 ± 0.012	2.0 ± 0.25	nd
*Echium italicum*	9.59 ± 1.96	0.204 ± 0.014	nd	0.138 ± 0.038	3.3 ± 0.9	nd	34.89 ± 10.44	0.135 ± 0.028	2.3 ± 0.57	0.076 ± 0.023
*Echium russicum*	7.41 ± 0.94	nd	0.478 ± 0.044	nd	5.1 ± 1.0	nd	10.85 ± 0.92	nd	8.6 ± 0.88	0.168 ± 0.096
*Echium vulgare*	0.91 ± 0.31	nd	0.329 ± 0.022	0.828 ± 0.288	3.4 ± 0.8	0.360 ± 0.025	0.61 ± 0.19	0.089 ± 0.019	1.3 ± 0.11	0.284 ± 0.183
*Lindelofia macrostyla*	11.81 ± 2.62	0.180 ± 0.002	0.379 ± 0.026	0.682 ± 0.206	7.7 ± 2.0	nd	11.98 ± 1.02	nd	9.8 ± 0.64	nd
*Lithospermum officinale*	2.36 ± 0.88	0.174 ± 0.003	0.754 ± 0.303	0.215 ± 0.017	1.2 ± 0.1	1.032 ± 0.06	0.81 ± 0.36	0.131 ± 0.015	1.8 ± 0.31	0.079 ± 0.002
*Nonea lutea*	0.99 ± 0.38	nd	nd	0.493 ± 0.077	7.7 ± 2.0	0.543 ± 0.07	0.60 ± 0.06	0.212 ± 0.035	2.4 ± 0.31	nd
*Pulmonaria mollis*	1.04 ± 0.22	nd	nd	nd	36.6 ± 1.2	nd	9.75 ± 0.25	nd	7.5 ± 0.24	nd
*Pulmonaria obscura*	2.37 ± 0.37	0.173 ± 0.003	nd	nd	15.6 ± 2.0	0.455 ± 0.02	13.14 ± 3.74	nd	7.9 ± 0.38	nd
*Omphalodes verna*	1.84 ± 0.52	nd	0.537 ± 0.076	nd	12.9 ± 2.7	0.675 ± 0.08	6.92 ± 1.92	0.105 ± 0.008	1.7 ± 0.15	nd
*Symphytum cordatum*	0.63 ± 0.17	nd	nd	nd	12.4 ± 1.3	nd	7.19 ± 0.75	0.144 ± 0.020	2.8 ± 0.22	nd
*Symphytum officinale*	9.38 ± 2.72	0.195 ± 0.010	nd	0.203 ± 0.055	4.5 ± 1.5	0.628 ± 0.32	25.77 ± 17.02	nd	7.1 ± 2.63	nd

PHBA: *p*-hydroxybenzoic acid; nd: not detectable.

Several species of the Boraginaceae family containing biologically active compounds were used in ancient medicine and are applied in modern pharmacology (Baczyńska & Lityńska-Zając 2005; Taravati et al. 2014). For example, due to its high allantoin content, the root of *S. officinale (Symphyti radix)* has been used for centuries as a poultice for treating wounds or burns and in treatment of rheumatism (Fell & Peck 1968). The results obtained confirmed that *S. officinale* was rich in allantoin; however, even higher allantoin concentrations (approx. 3.5%) were found in the roots of *E. italicum*. As a heterocyclic nitrogen compound, allantoin plays a role in nitrogen metabolism in plants (assimilation, transport). Moreover, its role in the chemical interactions between plants and other organisms, including insects and microorganisms, has been shown (Wang et al. 2012 and references therein).

Some earlier reports indicated presence of rosmarinic acid in Boraginaceae plants (Petersen et al. 2009; Fedoreyev et al. 2012). Antioxidative, astringent, antibacterial and anti-inflammatory activities have been described as the main biological activities of this compound (Petersen et al. 2009). The multitude of applications of rosmarinic acid for medical purposes has increased the interest in the possibilities of isolation of the compound from natural sources (Fedoreyev et al. 2012). Our results indicated that both *Anchusa* species contained approx. 2.5% of rosmarinic acid in the shoots, and its concentration in the shoots of *P. mollis* and *B. purpurocaerulea* exceeded 3.3%. Additionally, a high concentration (approx. 2.7%) of this compound was found in the roots of *A. undulata*. Moreover, the shoots of *C. minor, O. verna, P. obscura* and *S. cordatum* could also be a good source of rosmarinic acid.

Although some authors reported the presence of rutin in the *Anchusa* genus (Kuruüzüm-Uz et al. 2013), to the best of our knowledge, the present paper is the only report showing extraction and quantification of rutin as well as rosmarinic acid and some phenolic acids (*p*-hydroxybenzoic acid, hydrocaffeic acid, chlorogenic acid) in several Boraginaceae species. Among these compounds, rutin exhibits significant biological activities such as anti-oxidant, anti-inflammatory, anti-diabetic and anti-adipogenic effects (Chua 2013). Of the 17 species studied, eight contained rutin in the shoots. Six species were characterized by the presence of *p*-hydroxybenzoic acid in the shoots, 10 species (shoots or roots) contained hydrocaffeic acid, and chlorogenic acid was detected in the shoots of seven Boraginaceae species. However, the content of these compounds was in general relatively low – below 0.1%.

A characteristic feature of some Boraginaceae species is the presence of a red pigment shikonin and its derivatives in the roots (Papageorgiou et al. 1999; Chen et al. 2002). This naphthoquinone pigment was previously isolated from several Boraginaceae plants, for example: *Lithospermum erythrorhizon* Siebold & Zucc, *Arnebia euchroma* (Royle) I.M. Johnst, *Arnebia guttata* Bunge, *Onosma paniculatum* Bureau & Franch, *Onosma hookeri* C.B. Clarke (Chen et al. 2002 and references therein), *E. vulgare* and *E. russicum* (Dresler et al. 2015). Several therapeutic effects of shikonin and its derivatives were previously noted, including antitumor, anti-inflammatory and antimicrobial activities (Chen et al. 2002 and references therein). Additionally, its influence on the wound healing process was observed (Papageorgiou et al. 1999). Among the 17 Boraginaceae species studied, shikonin was extracted in a detectable concentration from three species belonging to the genus *Echium* (*E. vulgare, E. russicum, E. italicum*) and from *L. officinale.* It should be noted that the presented values represent the sum of shikonin derivatives since shikonin in the Boraginaceae plants usually occurs in many derivative forms (Papageorgiou et al. 1999). The HPCE technique used in the present study did not yield separation of shikonin derivatives; nevertheless, based on the comparison of the absorption spectra and retention time with the shikonin standard spectrum (similarity over 99.9%), a peak was identified and assigned as a sum of shikonin derivatives in the plant root extracts.

**Figure 1.**
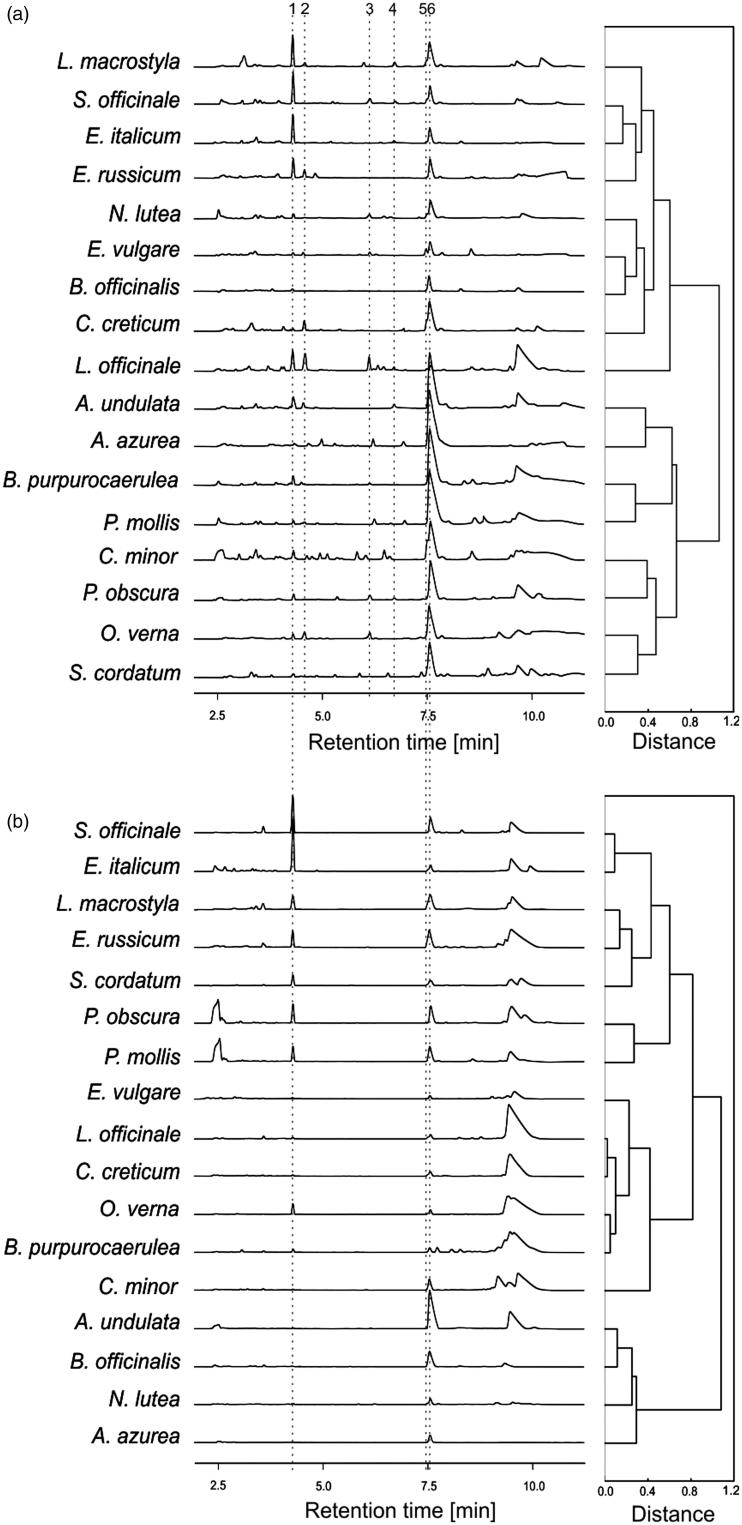
Common pattern of electrophoretic HPEC profiles (left side of the figure) and hierarchical clustering dendrograms of the phytochemical profiles (right side of the figure) of the shoots (a) and the roots (b) of 17 Boraginaceae species studied (*n* = 3). 1 – allantoin, 2 – rutin, 3 – chlorogenic acid, 4 – *p*-hydroxybenzoic acid, 5 – hydrocaffeic acid, 6 – rosmarinic acid.

Based on the phytochemical data, chemometric analyses were performed separately for the shoots and roots. Generally, two peaks of the two main compounds, allantoin and rosmarinic acid, together with some unidentified compounds eluting at 9–10 min had the greatest influence on the grouping process. The hierarchical cluster analysis (HCA) of the shoot data revealed species grouping in two main clusters (Figure 1(a)). One cluster consisted of eight species: *A. undulata, A. azurea, B. purpurocaerulea, P. mollis, C. minor, P. obscura, O. verna* and *S. cordatum.* This chemo-type generally grouped species with high concentrations of rosmarinic acid and a low concentration of allantoin. In the second cluster, two main subclusters were distinguished, i.e. one that grouped species accumulating relative high concentrations of allantoin in addition to rosmarinic acid: *L. macrostyla, S. officinale, E. italicum* and *E. russicum*, and the other one grouping species with a low concentration of allantoin: *N. lutea*, *E. vulgare*, *B. officinalis* and *C. creticum.* The analysis of the root data using the HCA method also successfully grouped the species into two main clusters; however, the distribution of the species was different from that for the shoot data (Figure 1(b)). The first cluster consisted of four species, *A. undulata, B. officinalis, N. lutea* and *A. azurea*, which exhibited no allantoin content and a moderate concentration of rosmarinic acid. In the second cluster, two subclusters were distinguished as well. The first chemo-type grouped seven species (*S. officinale, E. italicum, L. anchusoides, E. russicum, S. cordatum, P. obscura, P. molis*) generally accumulating high concentrations of allantoin and rosmarinic acid, while the other subcluster grouped six species (*E. vulgare, L. officinale, C. creticum, O. verna, B. purpurocaerulea,* and *C. minor*, which contained relatively low levels of allantoin and rosmarinic acid).

## Conclusions

Several biologically active compounds were identified and compared between 17 Boraginaceae species. The use of two modes of data acquisition (traditional by identification of several chemical markers as well as chemometric methods) showed the phytochemical profile of each species and allowed recognition of plants that could be a potential source of important biologically active compounds. For the first time, the report has shown extraction and quantification of several compounds (allantoin, *p*-hydroxybenzoic acid, rutin, hydrocaffeic acid, rosmarinic acid, chlorogenic acid, and shikonin) in Boraginaceae plants. Among the studied Boraginaceae species, some with potential high pharmaceutic activity have been distinguished. Besides *S. officinale*, which is commonly known as a source of allantoin, *E. italicum* appears to be another species that is rich in this compound (approx. 3.5% in the roots). Moreover, some species, e.g. *B. purpurocaerulea*, *P. molis*, *A. undulata* and *A. azurea*, could be good sources of rosmarinic acid for pharmacological purposes.
